# Mitotic Maturation Compensates for Premature Centrosome Splitting and PCM Loss in Human *cep135* Knockout Cells

**DOI:** 10.3390/cells11071189

**Published:** 2022-04-01

**Authors:** Zhenzhen Chu, Oliver J. Gruss

**Affiliations:** Institute of Genetics, Rheinische Friedrich-Wilhelms-Universität Bonn, Karlrobert-Kreiten-Str. 13, 53115 Bonn, Germany; s6zhchuu@uni-bonn.de

**Keywords:** SSX2IP, WDR8/WRAP73, CEP135, centrosome, pericentriolar material, microtubule organization, mitosis, spindle

## Abstract

Centrosomes represent main microtubule organizing centers (MTOCs) in animal cells. Their duplication in S-phase enables the establishment of two MTOCs in M-phase that define the poles of the spindle and ensure equal distribution of chromosomes and centrosomes to the two daughter cells. While key functions of many centrosomal proteins have been addressed in RNAi experiments and chronic knockdown, knockout experiments with complete loss of function in all cells enable quantitative analysis of cellular phenotypes at all cell-cycle stages. Here, we show that the centriolar satellite proteins SSX2IP and WDR8 and the centriolar protein CEP135 form a complex before centrosome assembly in vertebrate oocytes and further functionally interact in somatic cells with established centrosomes. We present stable knockouts of *SSX2IP*, *WDR8*, and *CEP135* in human cells. While loss of SSX2IP and WDR8 are compensated for, *cep135* knockout cells display compromised PCM recruitment, reduced MTOC function, and premature centrosome splitting with imbalanced PCMs. Defective *cep135* knockout centrosomes, however, manage to establish balanced spindle poles, allowing unperturbed mitosis and regular cell proliferation. Our data show essential functions of CEP135 in interphase MTOCs and demonstrate that loss of individual functions of SSX2IP, WDR8, and CEP135 are fully compensated for in mitosis.

## 1. Introduction

Microtubules (MTs) play crucial roles in in all eukaryotic cells throughout the cell cycle. In interphase, MTs organize the cytoplasm, helping to shape cell morphology and serving as transport routes for intracellular cargoes. In mitosis and meiosis (M-phase), however, tubulin dimers assemble into the highly dynamic bipolar spindle that drives chromosome segregation. Microtubule reorganization upon M-phase entry is facilitated by global alterations in the molecular composition of centrosomes, the major microtubule organizing centers (MTOCs) in animal cells [1].

Centrosomes generally comprise a pair of centrioles surrounded by layers of pericentriolar material (PCM) [2,3,4]. The PCM serves as a platform for proteins and protein complexes that govern MT nucleation and organization such as the γ-tubulin ring complex (γ-TuRC) [5]. The amount of γ-TuRC and other MT organizers in the PCM strongly increases at M-phase onset and enables overall increased MT assembly required for proper spindle formation.

Recently, we exploited *Xenopus* oocytes entering maturation (meiosis) to screen for novel factors required for MT functions in M-phase [6]. While vertebrate oocytes eliminate centrosomes during their development, the zygote forms a pair of new (zygotic) centrosomes from the paternal centriole (sperm basal body) and from a pool of maternally expressed centrosomal proteins that are already present in mature oocytes (eggs) immediately prior to fertilization. *Xenopus* egg lysates are, therefore, ideally suited for the identification of building blocks of centrioles and PCM. This fostered the identification of a previously uncharacterized centrosomal protein complex whose central moiety consists of two conserved proteins, Ssx2ip (synovial sarcoma X-breakpoint 2-interacting protein) and Wrap73/Wdr8 (WD repeat-containing, antisense to p73/WD repeat-containing protein 8; here referred to as WDR8/Wdr8) [6,7,8,9,10,11,12].

Ssx2ip, as a ubiquitously expressed 70 kDa α-helix-rich protein, localizes to centriolar satellites and maintains the nucleation capacity of centrosomes during embryonic cleavage divisions [6]. Substantial cell division defects are readily observed after downregulation of Ssx2ip in medaka embryos while defects are only partially reflected in knockdown of human somatic cells which display mild chromosome segregation errors [6].

In vertebrate somatic cells and embryonic cell extracts, Ssx2ip consistently interacts with a WD40 repeat protein, Wdr8. Orthologs of Wdr8 are highly conserved from yeast to man; frog and human sequences display 76% identity at the amino-acid sequence level [7,9,10,11]. Recent data show that the knockdown of WDR8 in human cells destabilizes SSX2IP and results in the dispersal of other centriolar satellite proteins including the pericentriolar material protein-1 (PCM-1). WDR8 knockdown yields measurable but non-engraving mitotic defects including compromised spindle positioning and spindle architecture [9,12]. In contrast, medaka knockout (ko) fish display strong chromosome segregation errors, abnormal timing of spindle formation, defects in its architecture, and impaired centrosome assembly, finally leading to early embryonic death after the loss of maternal Wdr8 [13]. In human somatic cells, WDR8 has been shown to interact with the centriolar protein CEP135 [10,11], while Cep135 expression and interaction with Wdr8 in embryonic systems have not been established.

Here, we show that Wdr8 interacts with newly expressed Cep135 in mature *Xenopus* oocytes before zygotic centrosome reformation. We further address essential functions of CEP135, WDR8, and SSX2IP in human somatic cells and generate homozygous KOs of all genes in human RPE-1 cells. Unlike early embryos, somatic cells tolerate individual gene losses although *cep135* KO cells show premature centrosome splitting and reduced PCM recruitment even in early interphase. Compromised PCM recruitment is, however, fully compensated for upon mitotic centrosome maturation, leading to normal spindle formation and function. Our data exemplifies how somatic cells counterbalance the loss of centrosomal protein functions via dominant mechanisms of mitotic PCM assembly. We propose that somatic cells can uncouple mitotic centrosome maturation from molecular requirements of PCM integrity in interphase.

## 2. Materials and Methods

### 2.1. Antibodies

A *Xenopus laevis* (xl) Wdr8 antibody was generated in rabbits against the C-terminal peptide sequence CYLDTEEEKK (Seramun Diagnostica GmbH, Heidesee, Germany) and used for immunoprecipitation. Antibodies against human (hs) WDR8 were generated in rabbits against the N-terminal (MNFSEVFKLSSLLCK) and C-terminal (ETEAVVGTACRQLGGHT) peptides (Seramun Diagnostica GmbH, Heidesee, Germany) and used for immunofluorescence (1:50). *Xenopus* Cep135 antibodies were generated in rabbits against N-terminal (AERKFINLRKRLDQLGYKQ) and C-terminal peptides (PERSILRTADRDGDRS, AEKSVSFKE) (Peptide Specialty Laboratory, Heidelberg, Germany). Antibodies against SSX2IP, guinea pig, IF (1:200); rabbit (rb), WB (1:400); rb PCM1, IF (1:500), WB (1:300), and rb anti-γ-Tubulin, IF (1:1000); were described previously [6].

The following commercial antibodies were used: rb anti-CEP135, Proteintech (part of Thermo Fisher Scientific, Planegg, Germany), 24428-1-AP (using CEP135 N terminus as antigen), IF (1:1000); rb anti-CEP135, Abcam (Cambridge, UK), ab75005 (using CEP135 C terminus as antigen), IF (1:1000); mouse (ms) anti-αTubulin, Sigma (Schnelldorf, Germany), T9026, IF (1:4000), WB (1:4000); ms anti γ-Tubulin: Sigma_Aldrich (Schnelldorf, Germany), T6557, IF (1:1000), WB (1:2000); rb anti-ARL13b, Proteintech (Planegg, Germany; part of Thermo Fisher Scientific), 17711-1-AP, IF (1:1000); rb anti-rootletin (CROCC), Novus Biologicals (Centennial, CO, USA), NBP1-80820, IF (1:200); rb anti-CNAP1, IF (1:300), Proteintech (part of Thermo Fisher Scientific, Planegg, Germany), 14498-1-AP; ms anti-CNAP1 for WB (1:750), Santa Cruz (Dallas, TX, USA), sc-390540; ms anti-NEK2 for WB (1:750), Santa Cruz (Dallas, TX, USA), sc-55601; rb anti-CDK5RAP2, Sigma-Aldrich (Schnelldorf, Germany), 06-1398, IF (1:600).

### 2.2. Preparation of Oocytes of Different Sizes 

Ovaries were dissected from female *Xenopus laevis* frogs and oocytes prepared as described previously [6]. The differently staged oocytes were separated using sieves that allowed separation of oocytes <120 μm, 120–300 μm, 300–560 μm, and 560–900 μm. Lysates were generated by passing through blue tips followed by centrifugation for 5 min at 3500 rpm, 4 °C. Isopropanol precipitation was used to extract total protein from crashed oocyte supernatant. Then, 4× Laemmli buffer was used to dissolve protein pellets. 

### 2.3. Xenopus Egg Extract Preparation, Immunoprecipitation, and Mass Spectrometry

The *Xenopus* egg extract preparation method was described previously [14]. MT assembly in the presence of 20 μM RanQ69L added to egg extract was used to test extract quality. For Wdr8 and Cep135 immunoprecipitation, 50 μg of xlWdr8 or xlCep135 antibody was pre-coupled to protein A magnetic beads (Thermo Fisher Scientific, Waltham, MA, USA) and added to 100 μL of egg extract. After half-hour incubation of antibody binding beads in egg extract on ice, the beads were washed three times with CSF-XB, three times with CSF-XB plus 250 mM KCl and 0.3% Triton X-100, and two times with PBS + 0.05% Triton X-100, before being eluted in 25 μL of 0.5% SDS. 

### 2.4. Mass Spectrometric Sample Analysis

#### 2.4.1. LC–MS Analysis

Protein samples, enriched by affinity purification, were separated by SDS PAGE for about 3 cm. Each entire lane was cut into pieces while excluding the band for the heavy chain of the antibody. In-gel digest samples were reduced, alkylated, and incubated with trypsin for 6 h at 37 °C [15]. The reaction was quenched by the addition of 20 µL of 0.1% trifluoroacetic acid (TFA; Biosolve, Valkenswaard, the Netherlands), and peptides were extracted, concentrated in a vacuum centrifuge, and dissolved in 15 µL of 0.1% TFA. Nanoflow LC–MS^2^ analysis was performed with an Ultimate 3000 liquid chromatography system coupled to an QExactive HF mass spectrometer (Thermo-Fischer, Bremen, Germany). A volume of 5 µL was injected into a self-packed analytical column (75 um × 200 mm, ReproSil Pur 120 C18-AQ, Dr Maisch GmbH, Ammerbuch-Entringen, Germany) and eluted with a flow rate of 300 nL/min in an acetonitrile gradient (3–40%). The mass spectrometer was operated in data-dependent acquisition mode, automatically switching between MS and MS^2^. Collision-induced dissociation MS^2^ spectra were generated for up to 15 precursors with a normalized collision energy of 29%. 

#### 2.4.2. Database Search

Raw files were processed using Proteome Discoverer version 2.5 (Thermo Fisher Scientific, Waltham, MA, USA) for peptide identification and quantification. MS^2^ spectra were searched against the Uniprot *Xenopus laevis* database (UP000186698_8355.fasta downloaded Nov 2019) and the contaminants database provided together with the MaxQuant software (version 1.6.0.15, Martinsried, Germany) using the Andromeda search engine with the following parameters: carbamidomethylation of cysteine residues as fixed modification and acetyl (protein N-term), oxidation (M), and deamidation (Q,N) as variable modifications; trypsin/P as the proteolytic enzyme with up to two missed cleavages was allowed. The maximum false discovery rate for proteins and peptides was 0.01, and a minimum peptide length of seven amino acids was required. All other parameters were default parameters of MaxQuant. 

### 2.5. Cell Culture and Cell Treatment

Telomerase reverse transcriptase immortalized human retinal pigment epithelial cell line (hTERT RPE-1) cells were cultured in DMEM/F12 (Thermo Fisher Scientific, Waltham, MA, USA) supplemented with 10% FBS, 2 mM l-glutamine in a 5% CO_2_ 37 °C incubator. G418 (800 μg/mL) was added to screening stably transfected cells. Thymidine (2 mM) was used to synchronize cells in S-phase. To enrich cells in metaphase, cells were treated for 16–18 h with 2 mM thymidine and then released for 8.5–9 h. To arrest cells in G_1_/G_0_, they were cultured for 2 days in medium without FBS. To monitor intercentrosomal distance in *cep135* KO upon MT depolymerization, 33 µM nocodazole was added for 1 h.

### 2.6. Generation of RPE-1 CRISPR KO Cell Lines

All CRISPR KOs were done by cloning guide RNAs to eSpCas9(1.1) (Addgene plasmid 71,814) or eSpCas9(1.1)-Puro (Addgene Plasmid 117,686). Guide RNAs were selected according to the Zhang Lab website: http://crispor.tefor.net/ (accessed on 1 October 2018) (*SSX2IP*), 1 July 2019 (*WDR8*), 1 September 2019 (*CEP135*). T7 endonuclease was used to check Cas9 cutting efficiency. The most efficient guide RNAs were used to generate stable KO cell lines. The following gRNA sequences were used: WDR8 gRNA1.2 (GGCGGCCATGAACTTCTCCG), WDR8 gRNA3 (TTGAGAATGTGGCGCCCGTC), WDR8 gRNA5 (GGACGGCCGCTACATGGCGC), CEP135 gRNA2 (G-ACAGTGGAGTGTTTACCTT), SSX2IP gRNA2 (GAGTATCTCATATCTTGATC), and SSX2IP gRNA3 (GCGGAAGAACCTTCTAGCTC). RPE-1 cells were transfected with a Neon Transfection System (Thermo Fisher Scientific, Waltham, MA, USA). Immunofluorescence, immunoblotting, and gene sequencing were used to confirm KO cell lines; *wdr8* ko308, *ssx2ip* ko2-13, and *cep138* ko8 were chosen as representative clones and used for all experiments unless otherwise indicated.

### 2.7. Generation of Stable CEP135 Rescue Cell Lines

The hs*CEP135* full-length cDNA (BC136535) was purchased from Biocat, Heidelberg, Germany. The *CEP135* full-length cDNA sequence encoding amino acids 1 to 1141, the *CEP135* N-terminal cDNA from amino acids 1 to 658, and the C-terminal cDNA from amino acids 648 to 1140 fused to AKAP450 PACT were cloned into the pEGFP-C1 plasmid (Clontech, TaKaRa Bio). The plasmids were transfected with Lipofectamine 2000 (Thermo Fisher Scientific, Waltham, MA, USA). 

### 2.8. Flow Cytometry

For cell cycle analysis by flow cytometry after propidium iodide staining, 10^5^ RPE-1 cells were seeded in 6 cm plates. When cells were close to 50% confluent, they were fixed with ice-cold 70% methanol overnight. The cells were stained with propidium iodide (PI 2.5 μg/mL, RNAase A 50 μg/mL) for 6 h. For each sample, more than 30,000 cells were counted. Data were analyzed with CFlow Plus software (Beckton Dickinson/BD Biosciences, version 1.0.264.15, San Jose, CA, USA).

### 2.9. Microtubule Regrowth Assay

A total of 1.5 × 10^5^ cells were seeded on a 3.5 cm plate with coverslips and grown for ca. 24 h. The plates were incubated on ice for 1 h to depolymerize microtubules. To regrow microtubules, coverslips were transferred to a new plate with prewarmed medium for 15 s and then fixed with 4%PFA/1% Triton X-100 by shaking for 20 min at room temperature. 

### 2.10. Indirect Immunofluorescence, Fluorescence Intensity Measurement, and Statistics

Cells on coverslips were fixed with 100% ice-cold methanol for 5 min unless otherwise indicated. After staining, coverslips were mounted in Fluoromount-G medium (Southern Biotech). Images for quantification were taken with an AxioPhot ZEISS or LSM880 confocal microscopy system (Zeiss Microscopy, Göttingen Germany). Quantification of fluorescence intensity was performed with Fiji Image J. For punctuated signals, the threshold was set to choose a proper area, and the intDen value was measured. For satellite protein signal intensity, a square of the indicated area was set around the γ-tubulin signal. *p*-Values were calculated using a two-tailed Student’s *t*-test, and significance levels were indicated as follows: * *p* = 0.01–0.05, ** *p* = 0.001–0.01, *** *p* = 0.0001–0.001, **** *p* < 0.0001. The R software (https://www.r-project.org/, accessed on 15 January 2021) and Adobe Illustrator 11.0 were used for generation of plots and typography. 

## 3. Results

**Figure 1:**
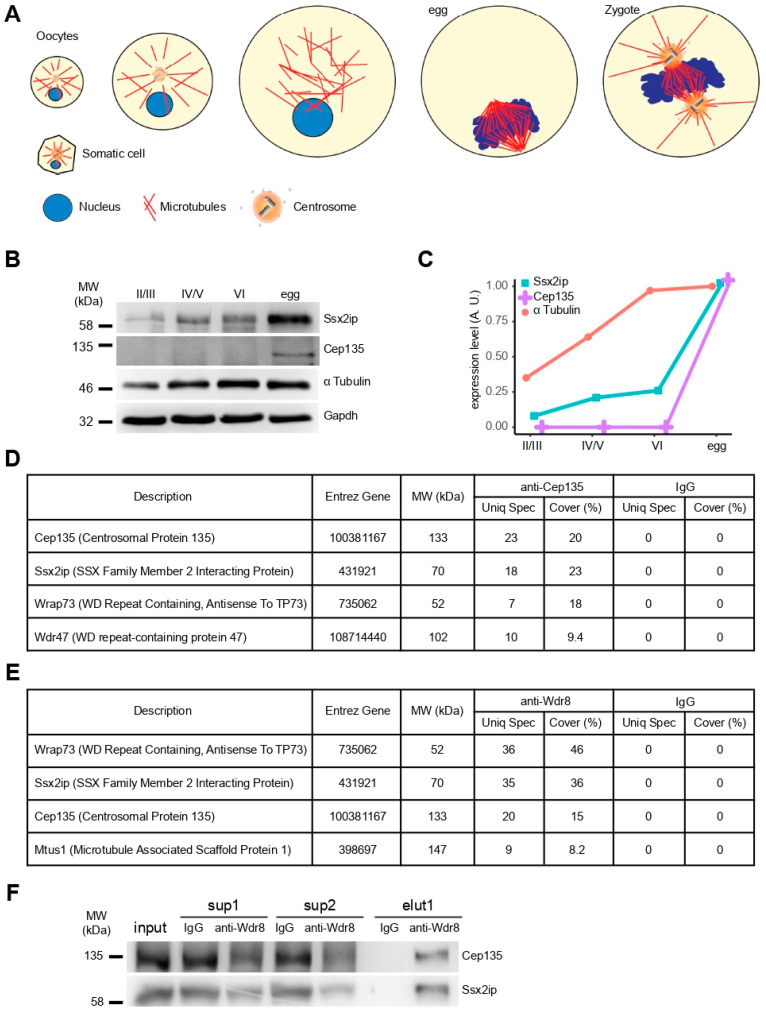
Interactome of Cep135, Wdr8, and Ssx2ip in *Xenopus* egg extracts. (**A**) Schematic drawing illustrating the state of centrosomes in somatic cells, developing oocytes, ovum (egg), and fertilized egg (zygote). (**B**,**C**) Immunoblotting (**B**) and corresponding quantification (**C**) of Ssx2ip, Cep135, and α-tubulin to evaluate protein expression at different *Xenopus* oocyte stages. The total amount of protein was controlled by glycerol aldehyde 3 phosphate dehydrogenase (Gapdh). (**D**,**E**) Identification of proteins specifically precipitated with antibodies against Cep135 (**D**) or Wdr8 (**E**) using tandem mass spectrometry. The list shows only a fraction of proteins identified. (**F**) Immunoprecipitations using antibodies against Wdr8 (elu1 Wdr8, elu1 IgG: control) analyzed by immunoblotting of Cep135 and Ssx2ip. Input (total extract) and supernatants (sup1/sup2) of extracts after IP are shown from two sequential precipitations.

To analyze if the expression regulation of Cep135 follows the elimination of centrosomes during oogenesis and their reassembly upon fertilization, we separated *Xenopus laevis* oocytes according to their size corresponding to different developmental stages (Figure 1A). Ssx2ip detection by Western blot confirmed increasing expression from immature (stage II) to fully grown (stage VI) oocytes, with a strong increase in fertilizable eggs (Figure 1B,C, Ssx2ip). Likewise, expression of Cep135 was only seen upon meiotic maturation in mature, nonfertilized eggs (Figure 1B,C, Cep135). This shows that both proteins are expressed in the preparation of fertilization and zygotic centrosome reassembly in maturing oocytes [16]. 

To investigate if both proteins interact, we immunoprecipitated (IP) Cep135 using specific antibodies directed against an N-terminal peptide of *Xenopus laevis* Cep135 from native *Xenopus* egg extracts and identified proteins in immunoprecipitates by tandem mass spectrometry. Mass spectrometry readily revealed Cep135 in IP samples using Cep135 antibodies but not in samples using unspecific IgGs (Figure 1D). Importantly, we also specifically detected Ssx2ip coprecipitating with Cep135, as well as the previously identified Ssx2ip interaction partner Wrap73/Wdr8 (Figure 1C). To further evaluate this interaction, we generated antibodies against frog Wdr8. Although the latter did not detect denatured Wdr8 in Western blot, mass spectrometry analysis of its IP fraction specifically showed Wdr8 itself, as well as Ssx2ip and Cep135 (Figure 1E). The latter IP fractions could also be detected by Western blotting to be specific interaction partners of Cep135 (Figure 1F). Taken together, this suggests that these three proteins build a stable complex in *Xenopus* egg extracts, most likely to prepare the egg cell for fertilization with the immediate need to rapidly assemble new centrosomes during cleavage divisions in early amphibian development. This is consistent with previous data from *Xenopus* egg extracts showing an interaction between Ssx2ip and Wdr8 [6,13], as well as data from human and mouse cells demonstrating that SSX2IP interacts with WDR8 and that WDR8, in turn, associates with CEP135 [10,11,17].

**Figure 2:**
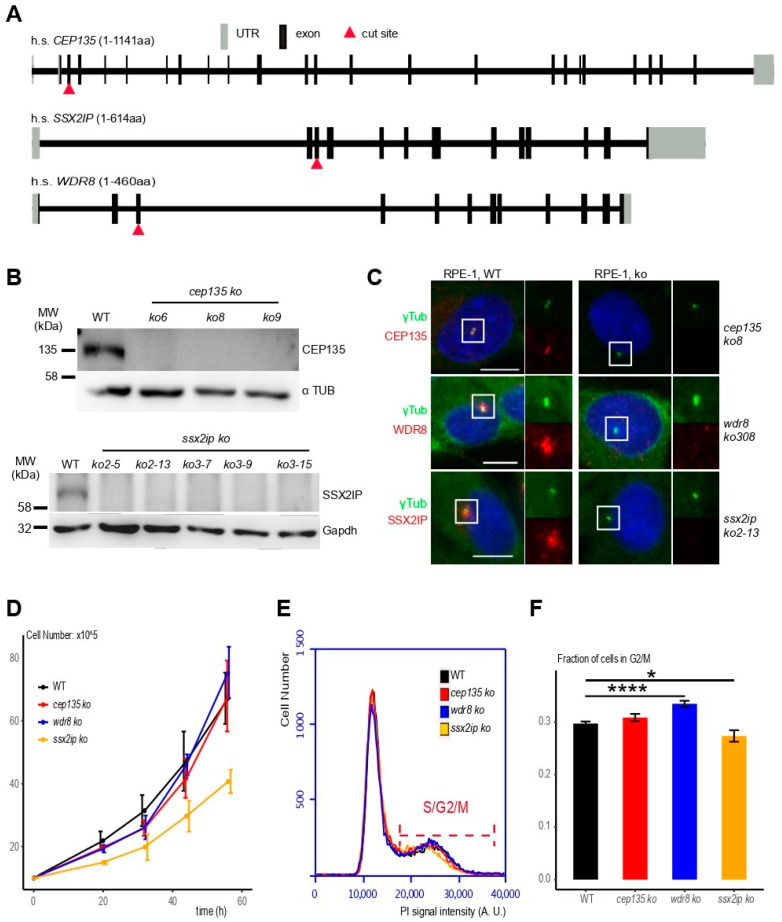
*cep135*, *wdr8*, and *ssx2ip* KOs generated by CRISPR/Cas9 in human RPE-1 cells line are vital and proliferate. (**A**) Genomic organization of human *CEP135*, *WDR8*, or *SSX2IP* loci with indicated targeting sites used for CRISPR/Cas9-mediated DNA cleavage. (**B**) Immunoblot to detect CEP135 (upper panel) or SSX2IP (lower panel) in cell clones after KO of the respective genes. Please note that the SSX2IP antibody tended to reveal unspecific signals in some immunoblots (see Figure 3A). Clones *wdr8* ko308, *ssx2ip* ko2-13, and *cep138* ko8 were used for all further experiments. (**C**) Immunofluorescence to detect CEP135, WDR8, or SSX2IP in human RPE-1 wildtype (WT) and representative *cep135*, *wdr8*, or *ssx2ip* KO cell lines (see Figures S1–S3 for all cell clones). (**D**) Cell proliferation in WT and *wdr8*, *ssx2ip*, and *cep135* RPE-1 KO cell lines. A total of 10^5^ cells (3.5 cm cm^2^ dish) were seeded and counted at indicated times (20 h, 32 h, 44 h, and 56 h). (**E**,**F**) DNA content analysis using propidium iodide (PI) staining to analyze cell-cycle progression in WT and *wdr8*, *ssx2ip*, or *cep135* RPE-1 KO cell lines. The indicated range shows cells in S/G_2_/M-phase. (**F**) Quantification of the fraction of cells in S/G_2_/M-phase. The graph shows average values from independent experiments ± S.D.; the *p*-value was calculated using a two-tailed Student’s *t*-test. * indicates *p* < 0.05, **** *p* < 0.001.

To better understand if and how SSX2IP, WDR8, and CEP135 functionally interact with each other in cells with established centrosomes, we generated individual KOs of all three open reading frames in human somatic RPE-1 (retinal pigment epithelial) cells using CRISPR/Cas9 gene targeting. Selected guide RNAs introduced DNA breaks in the third exons of the respective genomic human sequences (Figure 2A, red triangles; please note that 5′UTRs are also counted as exons). Sequencing of the loci revealed homozygotic loss-of-function mutations for *CEP135* (Figure S1), *SSX2IP* (Figure S2), and *WDR8* (Figure S3) in CRISPR KO cells (indicated in italics, lower case). Immunoblot analysis using antibodies against CEP135 and SSX2IP showed reduced signals in several clones of respective KO cells (Figure 2B, please note that WDR8 could not be detected by immunoblot). Consistently, immunofluorescence using specific antibodies against CEP135, WDR8, and SSX2IP revealed loss of the characteristic centriolar (CEP135) or satellite (SSX2IP, WDR8) signals in KO cells (Figure 2C; see Figure S4 for analysis of all cell clones). Despite the loss of these gene products, clones of KO cells could be readily identified and grew stably (Figure 2D). While *cep135* and *wdr8* KO cells even proliferated like untreated RPE-1 cells, *ssx2ip* KO cells showed a mild proliferation delay (Figure 2D, Figure S4; compare *ssx2ip* KO with controls and *cep135* and *wdr8* KO cells). When we further monitored cell-cycle progression, we found fewer *ssx2ip* KO cells in later cell-cycle stages (S/G_2_/M) than in controls and *cep135* KO cells, while more *wdr8* KO cells remained in late cell-cycle stages. Taken together, this shows that none of these gene products is individually essential for survival in non-transformed human RPE-1 cells, and that their complete loss of function leads to only marginal cell proliferation defects.

**Figure 3:**
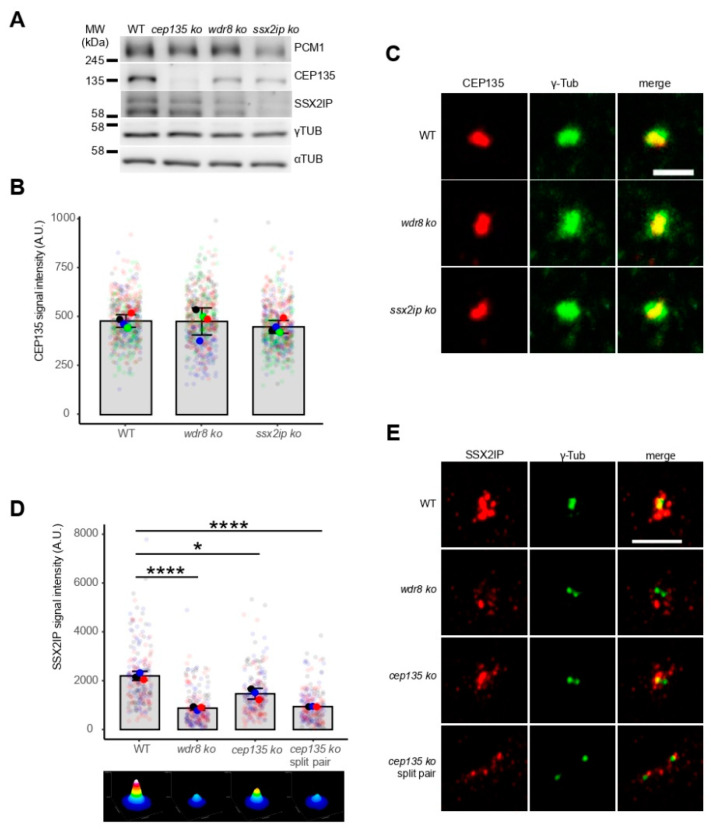
Protein expression and centrosomal localization of CEP135, WDR8, and SSX2IP are interdependent in RPE-1 cell lines. (**A**) Immunoblotting to show expression levels of PCM1, CEP135, SSX2IP, and γ-tubulin. α-Tubulin was used as a control for total protein loaded. Please note that the SSX2IP antibody tended to reveal unspecific signals in some immunoblots. (**B**–**E**) Centrosomal CEP135 (**B**,**C**) and SSX2IP (**D**,**E**) signals in WT and KO cells as indicated. *cep135* KO cells frequently displayed separated centrosomes with two distinct γ-tubulin signals (split pair). Representative immunofluorescence images (**C**,**E**, both with γ-tubulin signals) and quantifications (**B**,**D**). The ImageJ threshold feature was used to identify the region of the CEP135 signal in (**B**). A square (8.6 µm in *xy*) was drawn with the γ-tubulin signal in the center in (**D**). (**B**,**D**) Graphs show mean values (fat colored/black dots) of single experiments and individual data points (transparent dots). The added intensity of the two separated γ-tubulin signals (“split pair”) in *cep135* KO cells was quantified in (**D**). Three or four independent experiments were performed with more than 500 (**B**) or 210 (**D**) cells measured in each experiment. Errors bars show the SD from means; *p*-values were calculated with a two-tailed Student’s *t*-test. * indicates *p* < 0.05, **** *p* < 0.001. The surface plots represent sums (ImageJ *z*-projections) of all images. (**C**,**E**) Scale bars: 5 µm (**C**) or 2 µm (**E**).

To evaluate the interdependent centrosomal localization of all three proteins, we next monitored and compared their expression and subcellular distribution in wildtype and individual KO cells. Although expression of CEP135 was reduced in *ssx2ip* and *wdr8* KO cells (Figure 3A), its centrosomal localization remained unaffected (Figure 3B,C). In contrast, SSX2IP-containing centriolar satellites were seen at a significantly larger distance from centrosomes in *wdr8* and *cep135* KO cells (Figure 3D,E). Consistently, a more dispersed distribution of the centriolar satellite marker PCM1 was observed in *ssx2ip*, *wdr8*, and *cep135* KO cells (Figure S5). Of note, satellites were even more dispersed in frequently observed *cep135* KO cells with two separated centrosomes (Figure 3D,E, compare *cep135* KO with *cep135* KO split pair). This shows that satellites defined by SSX2IP or PCM1 require centrosomal localization of CEP135 to efficiently localize around centrosomes. In contrast, CEP135 localizes independently of SSX2IP and WDR8 to centrosomes, while its expression still relies on these two proteins. 

**Figure 4:**
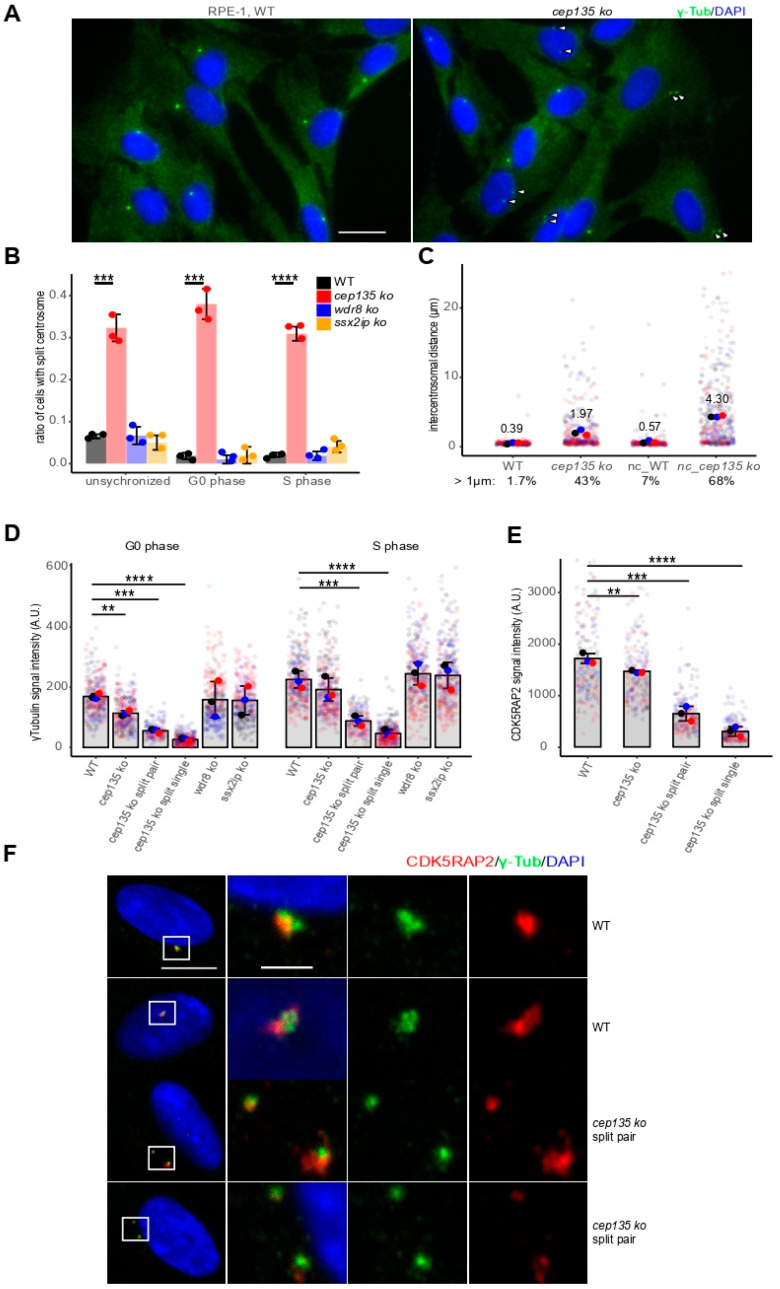
*cep135* KO cells show premature centrosome splitting and diminished PCM. (**A**) γ-Tubulin was stained to identify cells with premature split centrosomes and reduced PCM in WT and *cep135* KO cells. Scale bar: 20 µm. (**B**) Quantification of centrosome splitting by more than 1 µm at different cell-cycle stages in *cep135*, *wdr8*, and *ssx2ip* KO cell lines. (**C**) Quantification of centrosome distance in WT and *cep135* KO cells at G_1_ (48 h starvation) before and after nocodazole treatment that greatly increased centrosome distances in *cep135* KO cells. (**D**,**E**) Quantification of the centrosomal γ-tubulin (**D**) and CDK5RAP2 (**E**) signals in WT, *wdr8*, *ssx2ip*, and *cep135* KO cells. *cep135* KO cells displaying two distinct γ-tubulin signals were quantified separately either using summed intensities of both centrosomes (split-pair) or as separated values for each of the two individual centrosomes (split single). (**D**) Cells in G_0/1_ and S-phase. (**E**) G_0/1_ cells. (**B**–**E**) Graphs show mean values (fat colored/black dots) of single experiments and individual data points (transparent dots). Around 300 cells were measured for each cell line. Three independent experiments were performed. Error bars show the SD. The *p*-value was calculated with a two-tailed Student’s *t*-test. ** indicates *p* < 0.01, *** *p* < 0.005, **** *p* < 0.001. (**F**) Representative images showing γ-tubulin and CDK5RAP2 signals in WT and *cep135* KO cells. Scale bar: 10 µm or 2 µm (for enlarged areas).

To further investigate the frequently observed centrosome splitting in *cep135* KO cells (Figure 4A, arrowheads), we monitored and quantified cells with split centrosomes using γ-tubulin signals separated by more than 1 µm (Figure 4B) in WT and individual *cep135* KO cells at different cell-cycle stages. Of note, splitting occurred independently of the cell-cycle stage. Cells that were withdrawn from the cell cycle upon serum starvation and, thus, remained in G_0_/_1_ phase showed a similarly high number of split centrosomes as unsynchronized cells or cells that were blocked in S-phase using high thymidine concentration (Figure 4B). In contrast, split centrosomes remained rare in WT cells, as well as in *ssx2ip* and *wdr8* KO cells (Figure 4B). While the fraction of cells showing premature split centrosomes was lowest in control cells in G_0_/_1_ (1.7%), premature centrosome splitting increased to more than 43% in *cep135* KO cells and, after nocodazole treatment to depolymerize MT, even up to 68% (7% in WT controls Figure 4C). At the same time, nocodazole treatment increased the intercentrosomal distance from an average of 2.0 µm to 4.3 µm.

Centrosome splitting was previously observed as a consequence of reduced pericentriolar material [18,19,20]. To analyze if this was a cause for frequent, premature centrosome splitting in *cep135* KO cells, we quantified centrosomal signals of γ-tubulin, as well as CDK5RAP2, as markers of the PCM in *cep135* KO cells (Figure 4D,E). *cep135* KO cells showed significantly diminished γ-tubulin signals when cells were restricted to G_0_-phase upon starvation and, although to a lesser extent, to S-phase (Figure 4D, compare WT and *cep135* KO unspl). Centrosomal localization of CDK5RAP2 was significantly reduced in starved *cep135* KO cells compared to WT (Figure 4E, compare WT and *cep135* KO, Figure 4F). Importantly, both marker proteins were more dramatically reduced in *cep135* KO cells that had undergone centrosome splitting (Figure 4D–F, compare WT and *cep135* KO split single), albeit when the signal of the two separated centrosomes was summed (Figure 4D,E, compare WT and *cep135* KO split pair). 

**Figure 5:**
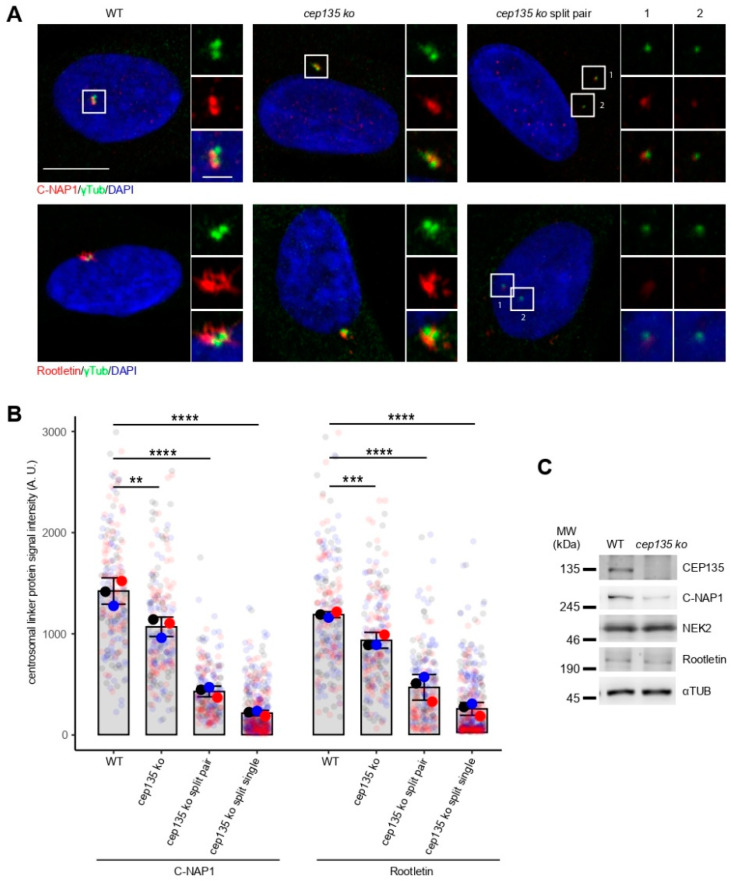
C-NAP1 and rootletin signals in prematurely split centrosomes of *cep135* KO cells. (**A**) Representative immunofluorescence images showing the signal of centrosome linker proteins C-NAP1 and rootletin in WT and *cep135* KO cells. *cep135* KO cells frequently displayed split centrosomes. Scale bar: 10 µm or 2 µm (insets). (**B**) Quantification of C-NAP1 and rootletin signal intensities in WT and *cep135* KO cells. Graphs show mean values (fat colored/black dots) of single experiments and individual data points (transparent dots) with error bars representing the SD from three independent experiments. *cep135* KO cells displaying two distinct γ-tubulin signals were quantified separately either using summed intensities of both centrosomes (split pair) or as separated values for each of the two individual centrosomes (split single). A total of 70 cells were quantified in each experiment. The *p*-value was calculated with a two-tailed Student’s *t*-test. ** indicates *p* < 0.01, *** *p* < 0.005, **** *p* < 0.001. (**C**) Immunoblotting showing protein expression of NEK2, C-NAP1, and rootletin. α-Tubulin was used as a loading control.

Previous publications have demonstrated the crucial role of PCM proteins directly functioning as centrosomal linker proteins (CLPs) in centrosome cohesion [21,22]. Therefore, we next examined whether loss of CEP135 may lead to reduced recruitment of CLPs, C-NAP1, and rootletin. When we visualized and quantified centrosomal rootletin and C-NAP1 by indirect immunofluorescence, we detected a significant loss of both proteins even in cells bearing a single centrosome, i.e., before centrosome splitting (Figure 5A,B). The reduction was stronger in cells that showed split centrosomes (Figure 5A,B). The total expression of C-NAP1 in cell lysates was diminished in *cep135* KO cells, while rootletin and the CLP-dissolving kinase NEK2A [23] remained at similar expression levels (Figure 5C). These data show that stable centrosomal loading of linker proteins rootletin and C-NAP1 is strongly reduced in a fraction of *cep135* KO cells, which may be due to reduced expression or stability of C-NAP1. Taken together, this suggests that the compromised physical linkage between centrosomes, as a consequence of compromised PCM establishment, precedes premature splitting in *cep135* KO cells. Our observation is consistent with previous reports based on RNAi knockdowns which showed that CEP135 is required for centrosomal targeting of C-NAP1 [20]. 

**Figure 6:**
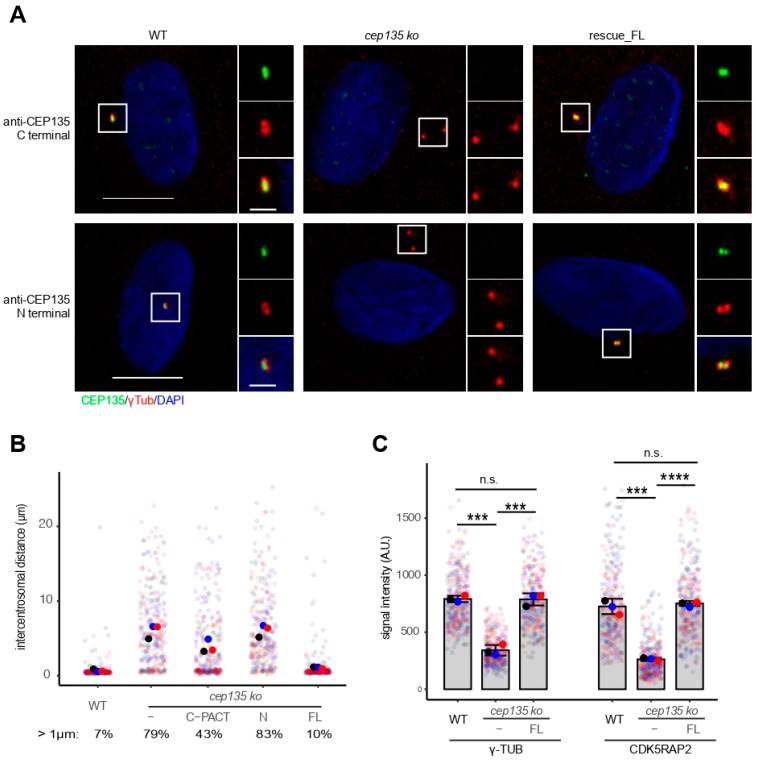
CEP135 expression rescues defects in *cep135* KO cells. (**A**) Representative immunofluorescence images showing exogenous CEP135 expression in *cep135* KO cells with antibodies against both N- and C-termini of CEP135. Scale bar: 10 µm or 2 µm (insets). (**B**) Centrosomal distance after nocodazole treatment in WT, *cep135* KO, and rescue cells stably expressing either full-length CEP135, the N-terminus domain of CEP135, or the C-terminus with the centrosomal targeting (PACT) domain of CEP135. (**C**) Quantification of γ-tubulin and CDK5RAP2 signals in WT, *cep135* KO, and rescue cells. (**B**,**C**) Graphs show mean values (fat colored/black dots) of single experiments and individual data points (transparent dots) with error bars representing the SD from three independent experiments (300 cells measured for each condition). The *p*-value was calculated with a two-tailed Student’s *t*-test. *** indicates *p* < 0.005, **** *p* < 0.001.

To prove that the observed centrosome splitting was solely due to the loss of CEP135, we generated *cep135* KO rescue cells by stably expressing full-length human *CEP135* cDNA fused to GFP. The frequently observed splitting of centrosomes was completely complemented when full-length CEP135 was expressed in *cep135* KO cells but not when we expressed the N-terminus of CEP135 alone, or to a much lesser extent, when cells expressed the C-terminal PACT domain of CEP135 (Figure 6A,B). This proves that the observed centrosome splitting defect in human cells was due to the loss of CEP135. Consistently, the observed reduction in PCM proteins γ-tubulin and CDK5RAP2 was fully restored in *cep135* KO cells expressing full-length human *CEP135* (Figure 6C).

**Figure 7:**
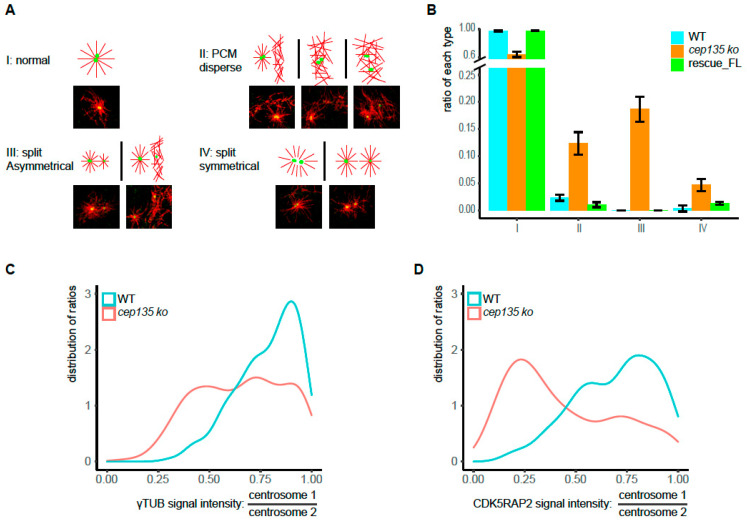
Imbalanced MTOC functions in *cep135* KO cells in interphase. (**A**) The typical patterns of MT regrowth in WT and *cep135* KO cells (red: MT, green: γ-tubulin). MT regrowth was allowed after cold treatment for 15 s. (**B**) Quantification of differently patterned microtubule structures in regrowth assays as exemplified in (**A**), I–IV. The graph shows mean values ± SD from three independent experiments. (**C**,**D**). The two distinct γ-tubulin and CDK5RAP2 signals in interphase cells with separated centrosomes were quantified, and their ratios (higher/lower value) were determined. The plot shows the distribution of ratios ranging from 1 (equal signals) to 0 (extreme asymmetry between the two signals). A total of 180 WT and 300 KO cells were measured.

We next rationalized that premature centrosome splitting and reduced PCM may lead to diminished MT nucleating functions in *cep135* KO cells. Using a MT regrowth assay, we tested centrosomal MT reassembly after MT depolymerization in the cold (Figure 7A) and quantified situations of expected “normal” MT asters (Figure 7A, I) and situations of dispersed MT organization centers (Figure 7A, II), split and asymmetrical MTOCs (Figure 7A, III), or two symmetrical MTOCs (Figure 7A, IV). While the number of abnormally arranged MT arrays was negligible in control and rescue RPE-1 cells, *cep135* KO cells frequently displayed abnormal MT organization (Figure 7B, please note that the frequency of all abnormal MTOCs (35%) matched the frequency of prematurely spilt centrosomes). In particular, dispersed MT organization (type II) was consistent with PCM decrease and instability. Considering the frequently forming asymmetric structures in MT regrowth assays, and the γ-tubulin and CDK5RAP2′s asymmetric distribution in *cep135* KO cells (Figure 4F), we further evaluated the imbalance of centrosomal proteins *cep135* KO cells with split centrosomes. The two distinct γ-tubulin and CDK5RAP2 signals in interphase cells with separated centrosomes were quantified, and their ratios (higher/lower value) were determined (Figure 7C,D). Indeed, the distribution of ratios in *cep135* KO compared to WT cells revealed a strong asymmetry in CDK5RAP2 signals and, to some extent, γ-tubulin signals (Figure 7C,D). 

**Figure 8:**
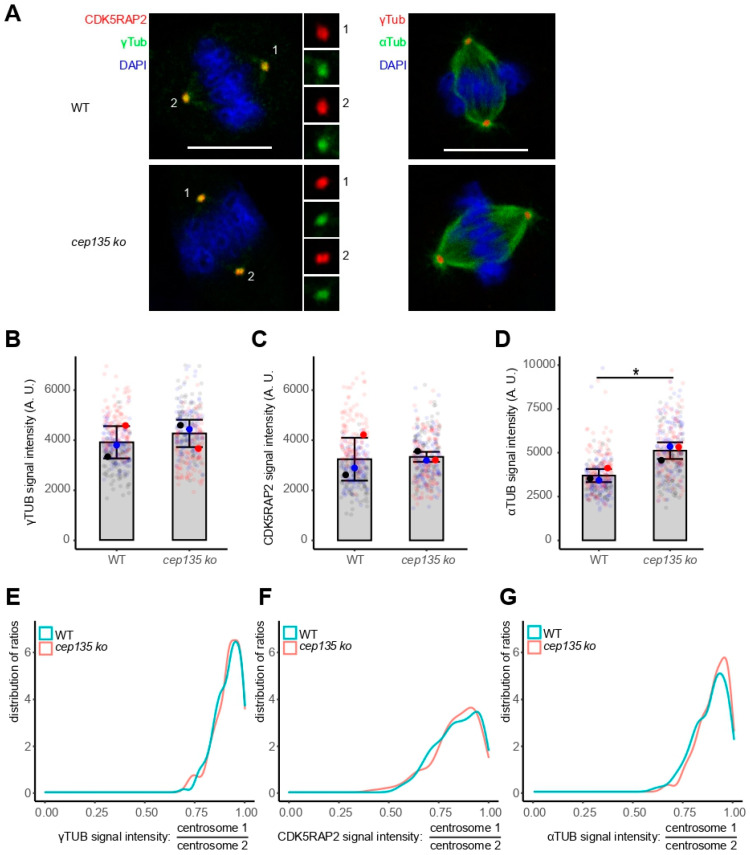
The two poles of metaphase spindles are balanced in *cep135* KO cells. (**A**) Representative images showing balanced spindle poles in metaphase. Scale bar: 10 µm. (**B**–**D**) Signal intensity of metaphase γ-tubulin (**B**), CDK5RAP2 (**C**), and α-tubulin (**D**) signals. A total of 150 cells were measured in total for each cell line. The graph shows mean values ± SD from three independent experiments. The *p*-value was calculated with a two-tailed Student’s *t*-test. * indicates *p* < 0.05. (**E**–**G**) The distinct γ-tubulin, CDK5RAP2, and α-tubulin signals of mitotic centrosomes/spindle poles were quantified, and their ratios (higher/lower value) were determined. The plot shows the distribution of ratios ranging from 1 (equal signals) to 0 (extreme asymmetry between the two signals). A total of 150 cells were measured in total for each experimental condition.

Given the decrease in and disorganization of PCM, as well as the high frequency of readily visible, abnormal MT regrowth arrays in *cep135* KO cells, we hypothesized frequent mitotic defects, although such a hypothesis was inconsistent with the normally proliferating *cep135* KO cells showing regular cell-cycle progression (Figure 2D–F). However, the analysis of mitotic spindle poles showed an apparently equal distribution of both γ-tubulin and CDK5RAP2 in individual cells (Figure 8A). In fact, the overall population of cells in mitosis showed an increased (although not significantly) accumulation of γ-tubulin and CDK5RAP2 (Figure 8B,C), in contrast to what we observed in interphase (Figure 7). Spindles formed in *cep135* KO cells displayed an even significantly higher MT density than in controls (Figure 8D). The distribution of γ-tubulin and CDK5RAP2 ratios between the two spindle poles showed high symmetry (8E and F), in agreement with the observed regular symmetry of spindle structures reflected by comparable MT signals in the two halves of the spindles (Figure 8G). 

Taken together, these data shows that *cep135* KO cells fully compensate for the previous loss of PCM and the asymmetric distribution of PCM proteins in prematurely split centrosomes entering mitosis. 

## 4. Discussion

Centrosomes generally serve as MTOCs in animal cells. MTOC functions are mostly localized in concentric layers of PCM recruited around centrioles. Stable centrosome cohesion in interphase holds two disengaged MTOC-competent centrosomes together as a single entity before centriole duplication. In late G_2_-phase, centrosome cohesion is relieved, and centrosomes separate to define the two poles (MTOCs) of the mitotic spindle. Balanced production of dynamic MTs from the two MTOCs in M-phase is essential for proper formation and function of the spindle. 

A set of a few conserved proteins turned out to be consistently essential in most metazoan species for centriole duplication: CEP192/SPD2, PLK4/ZYG1, SAS6, STIL/SAS5, and CPAP/SAS4 in humans. However, additional factors aid centriole stability, centrosome cohesion and splitting, the establishment of PCM around newly formed centrioles (centriole to centrosome conversion, CCC) starting in late mitosis, and the further increase (centrosome maturation) in PCM at mitotic onset. 

Previously, chicken DT40 cells were used to analyze essential functions of Cep135, a direct interaction partner of Sas-6, in a vertebrate model cell line [24]. Homozygous loss of *cep135* resulted in atypical centriolar structure without no obvious defects in centrosome composition and spindle formation. In contrast, knockdown of human CEP135 not only caused centriole duplication defects but strongly compromised spindle assembly and chromosome segregation, resulting in frequent mitotic failure [25]. More recently, high (“super”)-resolution light microscopy has greatly facilitated the analysis of individual centrosomal protein functions in human cells. Using 3D-SIM, as well as STED microscopy, Fu and colleagues could demonstrate that Bld10, the ortholog of human CEP135 in flies, precedes recruitment of Ana1/Cep295 and Asterless/Cep152 to establish centriole-to-centrosome conversion (CCC) [26]. Acting early in the assembly of a new (daughter) centriole, Cep135/Bld10 reaches out from the cartwheel structure beyond centriolar MT, enabling further protein interactions with Ana1 and Asl to refine daughter centrioles for the motherhood competent in the next cell cycle [27]. Analogously, CEP135, CEP295, and CEP152 have been shown to establish CCC in human cells [28]. This sets the ground for centriole duplication in the following S-phase. A picture emerges in which CEP135 is spotted as the communication point (“pinpoint” [29]) of the central cartwheel structure and the centriolar MT, stabilizes the centriole after cartwheel removal, fosters CCC, and improves competence for further duplication. 

In biochemical experiments, the WD40 repeat-containing protein WDR8 is the most consistently found interaction partner of CEP135 (https://thebiogrid.org/115018/summary/homo-sapiens/cep135.html, accessed on 8 March 2022). WDR8, in turn, communicates with the centriolar satellite protein SSX2IP. The latter proteins are essential for proper cell division in fish embryos, although their concerted function for centrosome structure remains unclear. Previous knockdown experiments in human cells indicated that acute knockdown of SSX2IP weakens MT anchoring to centrosomes, leading to reduced delivery of protein material required for proper centriole assembly [9,12]. In our experiments, we used human RPE-1 cells to establish homozygous KOs of *cep135* and of its interaction partners WDR8 and SSX2IP to analyze their functions in human somatic cells. All KOs produced viable and normally proliferating cells. KOs of *wdr8* and *ssx2ip* showed no detectable defects in centrosome appearance and mitotic progression, despite centriolar satellites being more dispersed in both KO cell lines, possibly as a consequence of weakened MT anchorage at MTOCs. In turn, *cep135* KO cells not only displayed dispersed centriolar satellites but showed diminished centrosomal targeting of γ-tubulin and CDK5RAP2 as key molecular players of MTOC function. 

Previously published functions of both SSX2IP and WDR8 in somatic cells were mostly based on acute siRNA knockdown experiments in combination with rescues using exogenously expressed, siRNA-resistant variants [6,8,9,10,11,12,17]. CRISPR/Cas9-mediated KO approaches will select cells for compensatory actions that allow, if mechanistically possible, stable proliferation despite complete loss of function of individual gene products. Given that nonredundant, essential functions cannot be compensated for, we conclude that gene products of CEP135, WDR8, and SSX2IP are not individually essential in human somatic cells in culture. This is in sharp contrast to the abovementioned RNAi experiments in the same (RPE-1) cells suggesting that loss of function of these genes forces cells to develop compensatory mechanisms that will finally allow regular proliferation of cells in culture. Compensation is most likely not possible, however, in developing vertebrates (medaka), where both knockdown and KO of *Ssx2ip* and *Wdr8* resulted in embryonic lethality after perturbed mitotic functions and chromosome segregation errors [6,13]. 

Despite possible compensatory mechanisms in somatic KO cells, RPE-1 *cep135* KOs displayed frequent centrosome cohesion defects, most likely caused by a general defect in PCM assembly. Diminished PCM in *cep135* KO cells included reduced recruitment of the linker proteins C-NAP1 and rootletin. According to siRNA knockdown experiments in human cells, C-NAP1 has previously been shown to interact directly with CEP135 and to be required for its anchoring in the PCM [20,30]. KO of *c-nap1* in human cells produced similar premature centrosome splitting to KO of *cep135* [31]. Our KO experiments, therefore, show that CEP135 is essential for interphase PCM assembly and that its function here cannot be compensated for. Human cells without CEP135 not only show loss of PCM and premature centrosome splitting, but also display a strong imbalance in PCM distribution between the split centrosomes. This results in defects of the interphase MT array that can be explained by misfunction of the MTOC. The overall reduced PCM and imbalance indicate that CCC is strongly compromised in *cep135* KO cells. Although we did not further investigate MT-related functions in interphase, we consider it likely that *cep135* KO cells show defects in Golgi organization and migratory behavior as previously observed for *c-nap1* KO cells [31]. 

Despite reduced and disbalanced interphase PCM, *cep135* KO cells assemble mitotic PCM to a defined size at both centrosomes and progress through mitosis with balanced spindle poles, with symmetrical spindle structures, and without further delay. Not only do they show balanced distribution of γ-tubulin and CDK5RAP2, but they also regain these PCM proteins to levels that even go beyond levels of these proteins on the spindle poles of mitosis in control RPE-1 cells. These data indicate that *cep135* KO cells compensate for the interphasic loss of PCM when entering mitosis. This may mean that even little residual interphase PCM suffices to seed PCM maturation in the mitosis of *cep135* KO cells. Alternatively, mitotic maturation may be largely independent of the preexisting PCM in interphase. Consistent with this, high-resolution microscopy visualized different forms of PCM organization in interphase and mitosis [1,32]. It remains to be investigated which molecular determinants, activated at M-phase onset, enable mitotic maturation in WT and cep135 KO cells. These may only be factors regularly involved in mitotic centrosome maturation or additional components that are activated upon loss of CEP135 and subsequent loss of PCM in interphase. 

Somatic cells may have evolved a surveillance mechanism that scans the functional size of the two MTOCs at the onset of M-phase in somatic cells and ensures balanced spindle pole activities despite unbalanced centrosomes before mitotic onset. Yet, after mitosis, centrosomes without CEP135 decompensate and fall back to structures with diminished PCM, as well as reduced MTOC functions. However, the history of the previous mitotic maturation may help centrosomes to remain, at least in part, functional during the next cell cycle. This may provide an explanation for residual PCM proteins in *cep135* KO cells and nonpenetrant centrosome splitting. It also explains the disbalance between, most likely, the older (mother) centrosome and the newly assembled centrosome with even lower levels of PCM in interphase. 

## Data Availability

Not applicable.

## Supplementary Materials

The following supporting information can be downloaded at https://www.mdpi.com/article/10.3390/cells11071189/s1: **Supplementary Figure S1. KO strategy for *cep135* in RPE-1 cells**. (A) Genome organization of human *CEP135* with the used guide RNA position indicated by the red arrowhead. (B) Sequencing results of *cep135* KO alleles of three different cell lines. (C) Immunofluorescence to document the loss of CEP135 in three different cell lines. *cep138* ko8 was used for all further experiments. Scalebar: 10 µm or 2 µm (insets); **Supplementary Figure S2. KO strategy for *ssx2ip* in RPE-1 cells**. (A) Genome organization of human *SSX2IP* with the used guide RNA positions indicated by the red arrowheads. (B) Sequencing results of *ssx2ip* KO alleles of five different cell lines. (C) Immunofluorescence to document the loss of SSX2IP in five different cell lines. *ssx2ip* ko2-13 was used for all further experiments. Scalebar: 10 µm or 2 µm (insets); **Supplementary Figure S3. KO strategy for *wdr8* in RPE-1 cells**. (A) Genome organization of human WDR8/WRAP73 with the used guide RNA positions indicated by the red arrowheads. (B) Sequencing results of *wdr8* KO alleles of three different cell lines. (C) Immunofluorescence to document the loss of WDR8 in three different cell lines. *wdr8* ko308 was used for all further experiments. Scalebar: 10 µm or 2 µm (insets); **Supplementary Figure S4. Proliferation analysis of ssx2ip KO clones**, 10^5^ cells (3.5 cm^2^ dish) were seeded and counted after 24 and 48 h (see Figure 2D); **Supplementary Figure S5. PCM-1 in WT cells, as well as *cep135, ssx2IP*, and *wdr8* KO cells**. (A) Quantification of PCM-1 intensities in WT and KO cells with *cep135* KO cells bearing a pair of split centrosomes (“split pair”). Graphs show mean values (fat colored/black dots) of single experiments and individual data points (transparent dots) with error bars representing the SD from three independent experiments with more than 100 cells quantified in each experiment. The surface plots represent sums (ImageJ *z*-projections) of all images. (B) Representative immunofluorescence images showing the signal of PCM-1 in WT cells, as well as *cep135, ssx2IP,* and *wdr8* KO cells. Scale bar: 10 µm.
