# Supplementary material for: Mitotic Maturation Compensates for Premature Centrosome Splitting and PCM Loss in Human cep135 Knockout Cells
Source: Cells. 2022 Apr 1;11(7):1189. doi: 10.3390/cells11071189 (PMC8997944; doi:10.3390/cells11071189)

Chu and Gruss, Supplementary Figure S1

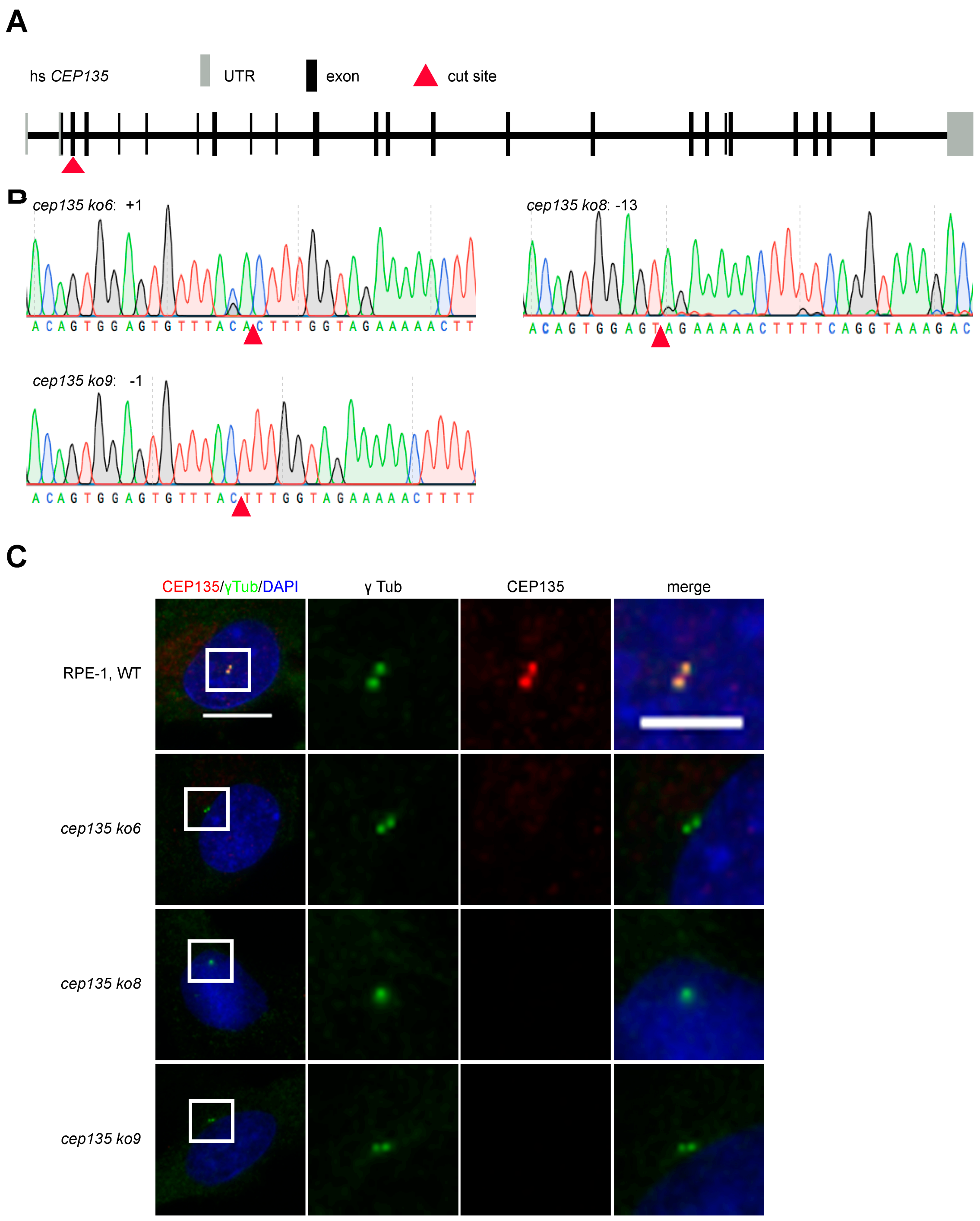

Chu and Gruss, Supplementary Figure S2

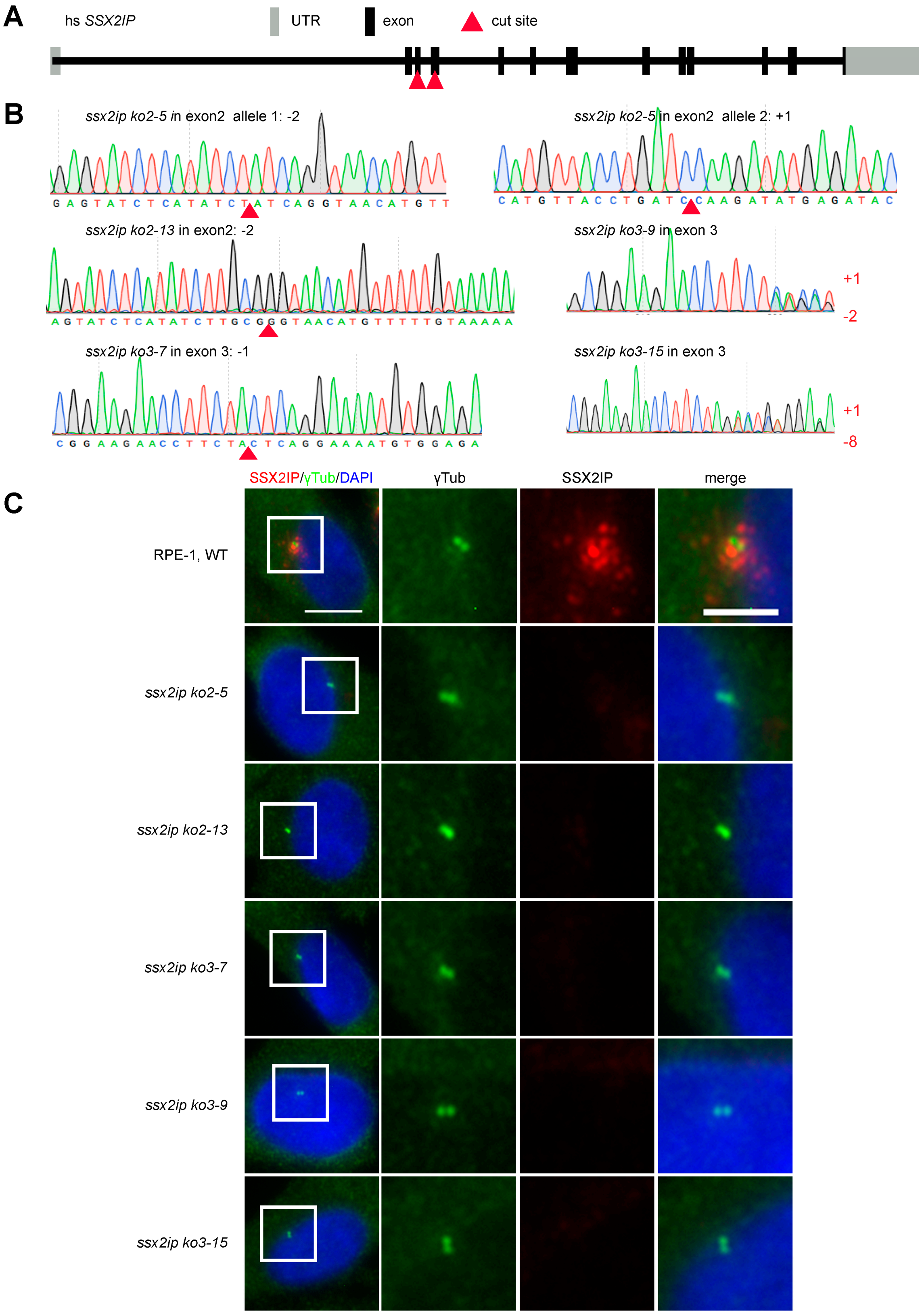

Chu and Gruss, Supplementary Figure S3

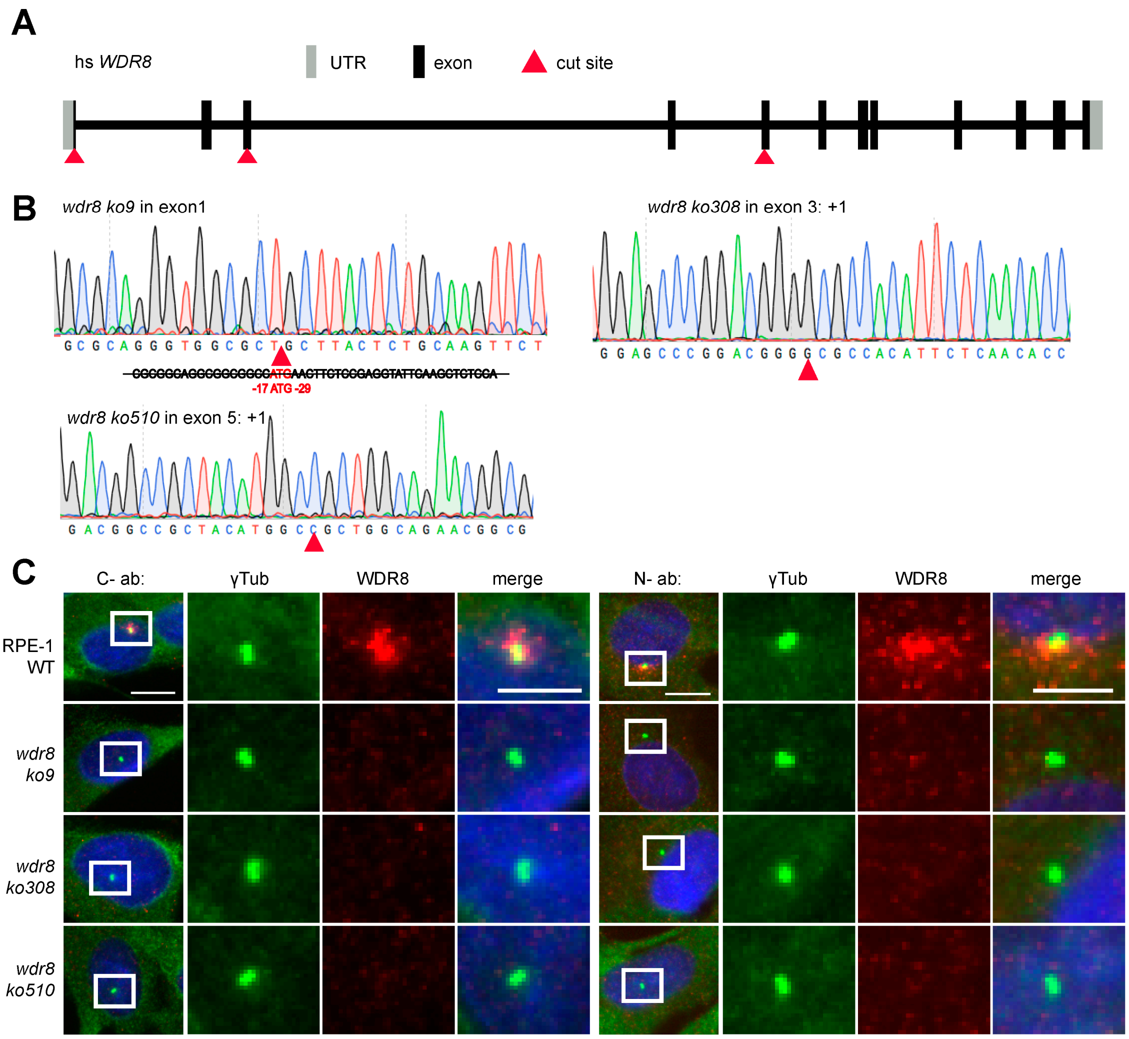

Chu and Gruss, Supplementary Figure S4

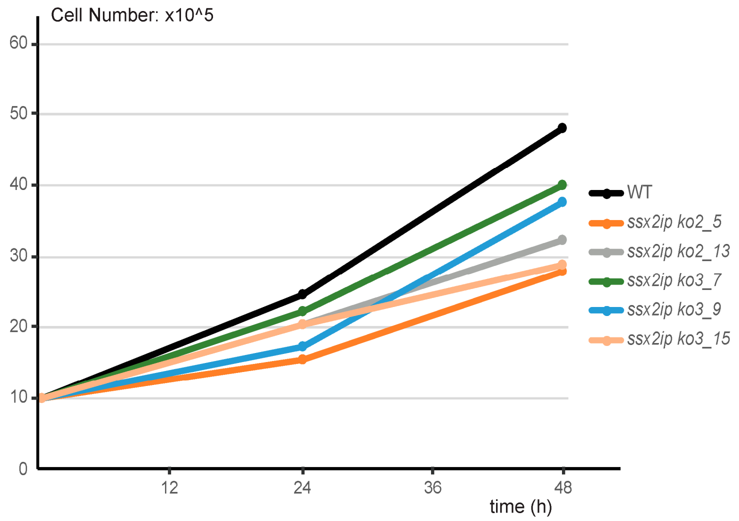

Chu and Gruss, Supplementary Figure S5

A

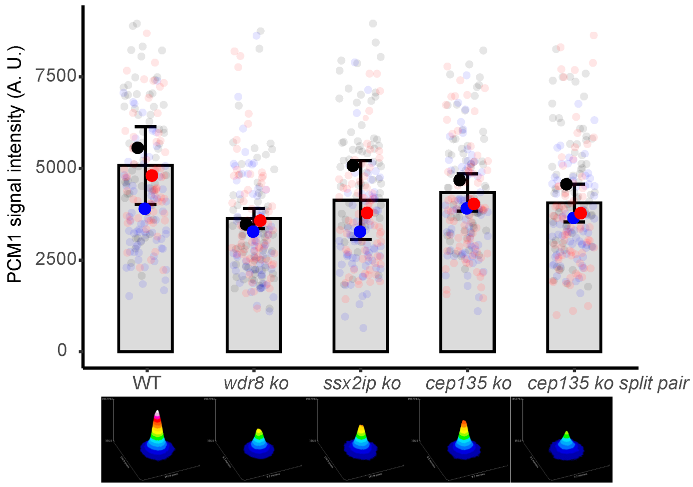

B

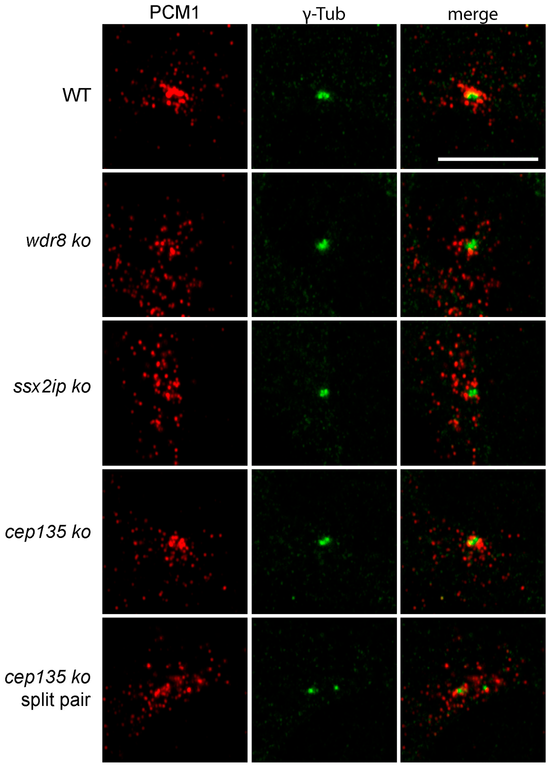

Supplement: Supplementary file 1 [file cells-11-01189-s001.zip › cells-1650685-supplementary.pdf]
